# Tlr2/4 Double Knockout Attenuates the Degeneration of Primary Auditory Neurons: Potential Mechanisms From Transcriptomic Perspectives

**DOI:** 10.3389/fcell.2021.750271

**Published:** 2021-10-25

**Authors:** Quan Wang, Yilin Shen, Yi Pan, Kaili Chen, Rui Ding, Tianyuan Zou, Andi Zhang, Dongye Guo, Peilin Ji, Cui Fan, Ling Mei, Haixia Hu, Bin Ye, Mingliang Xiang

**Affiliations:** ^1^Department of Otolaryngology and Head and Neck Surgery, Ruijin Hospital, Shanghai Jiao Tong University School of Medicine, Shanghai, China; ^2^Ear Institute, Shanghai Jiao Tong University School of Medicine, Shanghai, China

**Keywords:** hearing loss, spiral ganglion neurons, degeneration, Tlr2/4, transcriptome analysis

## Abstract

The transcriptomic landscape of mice with primary auditory neurons degeneration (PAND) indicates key pathways in its pathogenesis, including complement cascades, immune responses, tumor necrosis factor (TNF) signaling pathway, and cytokine-cytokine receptor interaction. Toll-like receptors (TLRs) are important immune and inflammatory molecules that have been shown to disrupt the disease network of PAND. In a PAND model involving administration of kanamycin combined with furosemide to destroy cochlear hair cells, Tlr 2/4 double knockout (DKO) mice had auditory preservation advantages, which were mainly manifested at 4–16 kHz. DKO mice and wild type (WT) mice had completely damaged cochlear hair cells on the 30th day, but the density of spiral ganglion neurons (SGN) in the Rosenthal canal was significantly higher in the DKO group than in the WT group. The results of immunohistochemistry for p38 and p65 showed that the attenuation of SGN degeneration in DKO mice may not be mediated by canonical Tlr signaling pathways. The SGN transcriptome of DKO and WT mice indicated that there was an inverted gene set enrichment relationship between their different transcriptomes and the SGN degeneration transcriptome, which is consistent with the morphology results. Core module analysis suggested that DKO mice may modulate SGN degeneration by activating two clusters, and the involved molecules include EGF, STAT3, CALB2, LOX, SNAP25, CAV2, SDC4, MYL1, NCS1, PVALB, TPM4, and TMOD4.

## Introduction

Sensorineural hearing loss (SNHL), the most common sensory deficit in the world, affects nearly 300 million individuals and costs 980 billion USD annually. SNHL mainly arises from the damage or death of auditory hair cells (Liu et al., 2016; Cheng et al., 2019; Gao et al., 2019; Han et al., 2020; Zhang S. et al., 2020, p. 1; Zhang Y. et al., 2020) and spiral ganglion neurons (SGN) (Guo et al., 2016, 2019, 2020; Liu W. et al., 2019; Ding et al., 2020; Liu et al., 2021; Yuan T. F. et al., 2021). These cells can be damaged by environmental insult (such as overexposure to loud sounds or exposure to aminoglycoside antibiotics or chemotherapeutics) (He et al., 2017, 2020, 2021; Qi et al., 2019; Jiang et al., 2020; Zhong et al., 2020; Zhou et al., 2020) or by genetic factors (Wang et al., 2017; Liu Y. et al., 2019; Qi et al., 2020, p. 4; Qian et al., 2020; Cheng et al., 2021; Fu et al., 2021; Zhang et al., 2021). As these mature cells lack the capacity for self-repair, the damage is permanent (Géléoc and Holt, 2014; Cheng et al., 2019; Tan et al., 2019; Zhang S. et al., 2020, p. 1; Chen et al., 2021).

Researchers have confirmed that many environmental insults immediately and directly damage hair cells, but the resulting SGN degeneration is chronic because of a lack of neurotrophic factors and peripheral stimuli (Kujawa and Liberman, 2009; Makary et al., 2011; Wu et al., 2021). The degeneration of the SGN leads to the loss and/or distortion of auditory information in the brain. Even as little as 10% of neural tissue degeneration can lead to a disproportionate change in the stimulation profile of the auditory nerve (Sriperumbudur et al., 2020). Spiral ganglion neurons would mainly have Ic subtype loss if the loss of peripheral stimulus occurs, which is similar to SGN aging (Shrestha et al., 2018). The degeneration of SGNs could induce distinct forms of plasticity in cortical excitatory and inhibitory neurons that culminate in net hyperactivity, increased neural gain, and reduce the adaptation to background noise (Resnik and Polley, 2021). On the other hand, once SGN degeneration occurs, cochlear implants will inevitably have poor performance. Histology of 12 temporal bones from 6 subjects indicated that higher residual SGNs could predict better performance after implantation in a given patient (Riss et al., 2008; Cusumano et al., 2017).

To elucidate the mechanism of SGN degeneration, a mouse model of rapid hair cell ablation with a single dose injection of kanamycin sulfate and furosemide (Taylor et al., 2008; Hu et al., 2017, p. 3; Ye et al., 2019), has frequently been used. In this SGN degeneration model, a single dose injection of kanamycin sulfate and furosemide immediately damaged hair cells but not SGNs (Gao et al., 2017). As auditory epithelia ablated, SGN, lacking neurotrophic factors and stimulus, will degenerate with increased lipofuscin area, damaged autophagic flux, and reduced density (Hu et al., 2017; Ye et al., 2019).

Tlr polymorphisms were correlated with hearing preservation in bacterial meningitis survivors (van Well et al., 2012). Koo et al. (2015) assumed that this kind of hearing preservation difference is associated with Tlr-mediated BLB permeability. Researchers have also found that Tlr4 knockout mice had better hearing function following noise trauma without affecting sensory cell viability under physiological conditions (Vethanayagam et al., 2016). De Paola et al. (2012) found that Tlr4 inhibition attenuates motor neuron degeneration both *in vitro* and *in vivo*. Tlr4 deficiency could also protect mice against ischemia and axotomy-induced retinal ganglion cells degeneration, and they assumed that better neuron preservation was associated with reduced parenchymal stress responses via the ERK, JNK, and P38 signaling pathways (Kilic et al., 2008). The innate immune response of the nerve microenvironment is involved in functional recovery during Wallerian degeneration, and Tlr2 and Tlr4 can regulate synaptic stability in the spinal cord after peripheral nerve injury (Freria et al., 2016). Researchers have found that systemic microbial TLR2 agonists could induce neuron degeneration in Alzheimer’s disease mice and direct CNS delivery of a selective TLR2 antagonist blocked the neurotoxicity of systemically administered zymosan, indicating that CNS TLR2 mediates this neuron degeneration process (Lax et al., 2020). Previous studies have also demonstrated that administration of anti-TLR2 alleviated α-synuclein accumulation in neuronal, neuroinflammation, neurodegeneration, and behavioral deficits in an α-synuclein tg mouse model of PD/DLB and proposed TLR2 immunotherapy as a novel therapeutic strategy for neuron degeneration (Kim et al., 2018). These studies suggest that Tlr molecules may regulate the degeneration pathogenesis of auditory systems, especially spiral ganglion neurons.

Although SGN degeneration is characterized by increased lipofuscin area, damaged autophagic flux, and reduced density, a comprehensive understanding of the SGN degeneration transcriptome landscape is still lacking, and the role of molecules such as Tlr that may regulate the pathogenesis remain unknown. Here, we analyzed the transcriptome landscape of SGN degeneration and identified that Tlr2 and Tlr4 (Tlr2/4) might regulate the pathogenesis of degeneration. Based on these findings, we analyzed the role of Tlr2 and Tlr4 in SGN degeneration and found that genetic deletion of Tlr2/4 in combination attenuates SGN degeneration in mice without interfering with classic Tlr signaling pathways. Furthermore, we used transcriptome analysis to identify the key modules and pathways. Identification of these specific molecules and pathways could potently facilitate the protection of the auditory system.

## Materials and Methods

### Animals

B6.B10ScN-Tlr4lps-del/JthJ and C.129(B6)-Tlr2^*tm*1K*ir*^/J mice (The Jackson Laboratory, Bar Harbor, ME, United States) were used for Tlr2/4 double knockout. Only 8-week-old male mice without a history of ototoxic damage or noise exposure were used for model establishment. The animals were housed under a standard 12-h light/dark cycle and were allowed free access to water and a regular mouse diet. The cochleae of each animal were collected and assigned to different assays to reduce animal usage and maintain sufficient sample sizes. The following genotyping primers were used to examine the Tlr2 knockout allele: 5′-CTTCCTGAATTTGTCCAGTACA-3′; 5′-GGGCCAGCTCATTCCTCCCAC-3′; 5′-ACGAGCAAGATC AACAGGAGA-3′ (mutation allele 334 bp, WT allele 499 bp). The following genotyping primers were used to examine the Tlr4 knockout allele: 5′-GCAAGTTTCTATATGCATTCTC-3′; 5′-CCTCCATTTCCAATAGGTAG-3′; 5′-ATATGCATGATCAA CACCACAG-3′; 5′-TTTCCATTGCTGCCCTATAG-3′ (mutated allele 140 bp, WT allele 390 bp). The procedures were approved by the Ethics Committee of Xinhua Hospital, affiliated with Shanghai Jiao Tong University School of Medicine (Shanghai, China).

### Spiral Ganglion Neurons Degeneration Model

Mice of the SGN degeneration group were subcutaneously injected with 1 g/kg kanamycin sulfate (Sigma-Aldrich, E004000), and intraperitoneally injected with 0.4 g/kg furosemide (Tianjin Pharmaceutical Group, H12020527; 10 mg/ml) after 30 min (Hu et al., 2017; Ye et al., 2019). Mice in the control group were simultaneously and intraperitoneally treated with equal doses of saline. The mice were sacrificed 7, 15, and 30 days after drug administration, and the temporal bones were removed and dissected in PBS at 4°C.

### Measurement of Auditory Brainstem Response

The mice were anesthetized with a ketamine (0.1 mg/g)/xylazine (0.01 mg/g) mixture. Body temperature was maintained at 37°C with a warming blanket. An active needle electrode was placed in the midline of the vertex of the skull, a reference electrode at the mastoid areas, and a ground electrode in the low back area. The ABRs were provoked with tone bursts of 4, 8, 11, 16, 22, and 32 kHz, generated by a D/A converter (RP2.1; TDT) and relayed to an attenuator (PA5; TDT), an amplifier (SA1; TDT), and a magnetic speaker (MF1; TDT). Mouse auditory brainstem responses were filtered (100–3,000 Hz), amplified, and averaged using TDT hardware and software. Responses were recorded from 90 dB SPL to 10 dB below the threshold level in 5 dB descending steps. The ABR threshold was defined as the lowest intensity that reliably elicited a detectable response. The average ABR threshold shifts between wild-type and DKO mice were compared using two-way ANOVA with the two factors group × frequency. If significant group effects were identified, the Welch two-sample *t*-test was used to evaluate each frequency between the two groups.

### Histopathological Analysis

The excised cochlea was immersed in 4% paraformaldehyde in phosphate-buffered saline solution for 12 h and decalcified in 10% EDTA for 5 days. Specimens were sliced into 4 μm sections, mounted onto silane-coated slides, stained, and observed under a light microscope. The evaluation of cochlear histology included the apical, middle, and basal regions in the Rosenthal canal and the organ of Corti. Every fifth modiolar section for a certain cochlea (four in total) was subjected to histopathological assessment. The same animals were used for the IHC staining. Primary antibodies against the following antigens were used: Caspase 3 (Abcam, ab44976), p65 (Abcam, ab32536), and p38 (Abcam, ab170099). For immunofluorescence staining, frozen sections were blocked with 10% donkey serum (Jackson, 017–000-121) and 0.5% Triton X-100 (Sigma-Aldrich, 9002–93-1) and then incubated with rabbit anti- Caspase 3 (Abcam, ab44976; 1:400) or p65 (Abcam, ab32536; 1:500) overnight at 4°C. After the sections were washed 3 times with PBS, donkey anti-rabbit Alexa Fluor 594 (Jackson, 711–585-152; 1: 500) were used to incubate samples at 37°C in the dark for 30 min. Finally, the sections were stained with DAPI (Beyotime, C1002; 1:2,000) for 5 min. For immunohistochemical staining, paraffin sections were heated at 67°C for 2 h and then deparaffinized in deparaffinized in xylene and rehydrated in alcohol. After the sections were washed 3 times with 0.1 M PBS, they were incubated with 3.0% H2O2 for 30 min at room temperature. The sections were then incubated with the primary p38 (Abcam, ab170099; 1:200) antibody overnight at 4°C. The secondary horseradish peroxidase-labeled goat anti-rabbit IgG antibody (Beyotime, A0208, 1:500) was added for 1 h, and finally, the sections were stained with 3,3N-diaminobenzidine (Beyotime, P0203). Unless otherwise stated, middle cochlear tissues (11–16 kHz region) were used for analyses.

### High Throughput Sequencing

Total RNA from Cochlear modiolus tissues was extracted using the QIAGEN RNA Extraction Kit (Cat. No.74104). Agarose gels (1.5%) were used to detect RNA degradation and contamination. Illumina Hiseq 2,500 and Hiseq 4,000 were used to characterize the transcriptome landscape of SGN following the manufacturer’s recommendations. The following procedures were performed using Hisat2 (version 2.1.0) to align the clean reads with the reference genome of mice (Kim et al., 2015) and StringTie (version 1.3.3b) to evaluate gene expression (Pertea et al., 2015).

### Functional Annotation

Gene set enrichment analysis (GSEA) (Subramanian et al., 2005; Yu et al., 2012) was used to extract biological insight from RNA-seq data. GSEA aims to determine whether members of gene set S tend to occur in the extremes (top or bottom) of a given list from RNA-seq results. The enrichment score (ES) indicates the degree of a set S overrepresentation at the extremes of the gene ranked list L. ES was calculated as follows:


(1) $$P_{h}\left(S,j\right)=\sum_{g_{j}\in S}\frac{{\left|r_{j}\right|}^{w}}{\sum_{g_{j}\in S}{\left|r_{j}\right|}^{w}}$$

(2) $$P_{m}\left(S,j\right)=\sum_{g_{j}\in S}\frac{1}{\left(N-N_{h}\right)}$$

(3) $$ES=max\left(\left|P_{h}-P_{m}\right|\right)$$

*P*-value was calculated by estimating the statistical significance of the ES by empirical phenotype-based permutation test procedures.

Terms used for functional annotation include Gene Ontology (Ashburner et al., 2000) and Kyoto Encyclopedia of Genes and Genomes (KEGG) (Kanehisa and Goto, 2000). Functional annotation of GO, including biological processes, cellular components, and molecular functions, and KEGG is a common omnibus to systematically interpret gene functions, facilitating an intensive understanding of genomic information and higher-order functional information.

### Protein-Protein Interaction and Module Analysis

The construction of PPI networks imported the Search Tool for the Retrieval of Interacting Genes database (STRING) (Szklarczyk et al., 2021), and was analyzed using Cytoscape (version 3.6.1) software (Shannon et al., 2003). STRING (version 11.0) covers 2,000 million interactions of 24.6 million proteins from 5,090 organisms. When fed with differentially expressed genes, STRING was able to identify the most significantly interactive associations, in which a criterion of a combined score > 0.4, was set as the significance level. Cytoscape plugin molecular complex detection (MCODE) can illustrate the core modules in the PPI network. The false degree cut-off, node score cut-off, haircut, false K-core, and maximum depth from seed were set at 2, 0.2, true, 2, and 100, respectively (Wang et al., 2018, 2020; Yan et al., 2019).

### Statistical Analysis

Computations and data visualization were performed using R 4.0.2. Distribution of the data was assumed to be normal, but this was not formally tested. No statistical methods were used predetermine sample sizes, but our sample sizes are like those reported in our previous publications (Hu et al., 2017; Ye et al., 2019). Statistical differences in auditory physiology, SGN counts were analyzed using two-way ANOVA, followed by Welch *t*-test. All statistical analysis results were expressed as the mean ± standard error of the mean (SEM). Statistical significance was defined as *P* < 0.05.

## Results

### Spiral Ganglion Neurons Degeneration Established by Cochlear Sensory Epithelial Ablation

**FIGURE 1:**
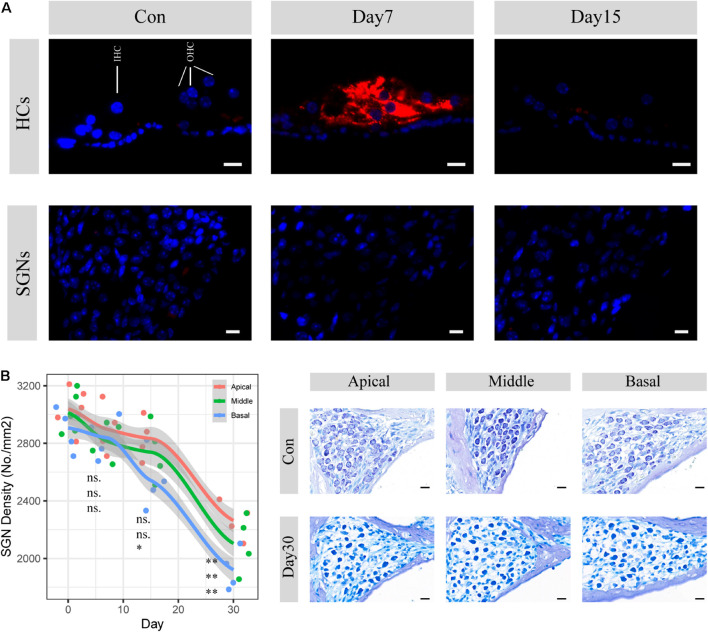
SGN degenerati on established by cochlear sensory epithelial ablation. **(A)** Representative images of CASP3 IF staining in hair cells and spiral ganglion areas. Hair cell apoptotic activities were significantly upregulated within 7 days but disappeared 15 days after injection of kanamycin and furosemide. There are no apoptosis activities seen in the SGN areas during the period. HCs: hair cells; SGNs: spiral ganglion neurons. Red: caspase3; Blue: DAPI. Scale bar: 10 μm. **(B)** SGN density evaluation 7, 15, and 30 days after the injection of kanamycin and furosemide. On the 30th day, SGN density significantly decreased at the apical, middle, and basal turn of the cochlea (apical: *N* = 9, *t* = 8.14, *P* < 0.01, middle: *N* = 9, *t* = 7.53, *P* < 0.01; basal: *N* = 9, *t* = 10.33, *P* < 0.01). Statistical significance of other time points was also denoted with the order of apical, middle and basal, respectively (ns: not significant; *P < 0.05; **P < 0.01.). Gray area indicated the standard error range calculated by loess method. Right panel shows the representative images of SGN before and 30 days after the injection of kanamycin and furosemide. Scale bar: 10 μm.

**FIGURE 2:**
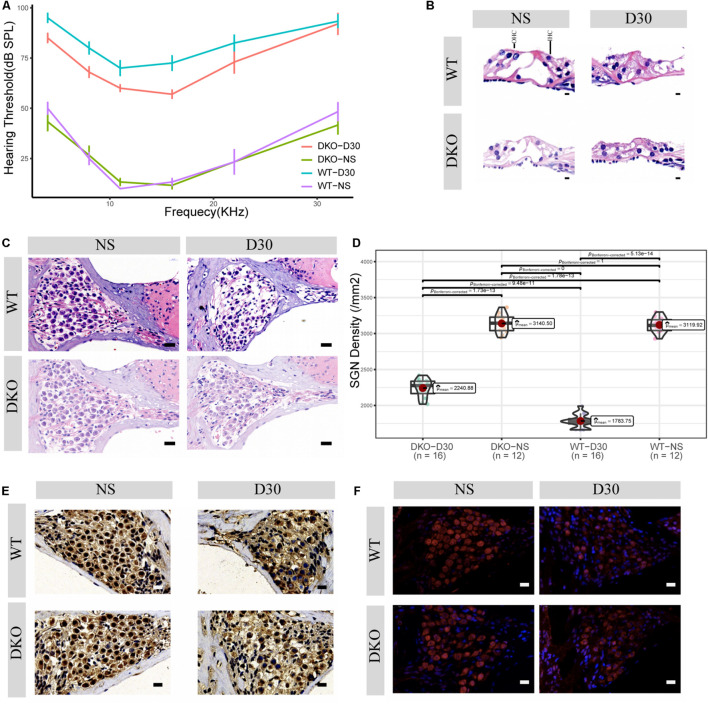
Physiological and morphological changes in Tlr2/4 DKO mice and WT littermates. **(A)** The hearing threshold of auditory brainstem response of WT and DKO mice in the SGN degeneration model. Mice maintained normal hearing threshold in the normal saline treated group (*N* = 8, *P* = 0.7148, *F* = 0.105, two-way ANOVA). On the 30th day after kanamycin and furosemide injection, DKO mice exhibited a hearing preservation advantage compared with WT mice (*N* = 13, *P* = 0.00446, *F* = 8.700, two-way ANOVA). **(B)** Hair cell loss of WT and DKO mice. Saline treated mice had normal hair cells. On the 30th day of kanamycin and furosemide injection, both DKO and WT mice lost all hair cells and the tunnel of Corti had collapsed. Scale bar: 10 μm. **(C)** SGN loss of DKO and WT mice. Mice maintained a normal SGN count in normal saline treated group (*N* = 24, *P* = 0.675, *t* = 0.43, Welch Two Sample *t*-test). On the 30th day of kanamycin and furosemide injection, DKO mice have higher SGN density compared with WT mice Scale bar: 10 μm. **(D)** Statistics of panel C. On the 30th day of kanamycin and furosemide injection, DKO mice have higher SGN density compared with WT mice (*N* = 32, *P* < 0.001, *t* = 11.471 Welch Two Sample *t*-test). **(E)** Representative IHC image of p38 expression of SGN in WT and DKO mice. Scale Bar: Scale bar: 10 μm. **(F)** Representative IHC image of p65 expression of SGN in WT and DKO mice. Scale Bar: Scale bar: 10 μm.

SGN degeneration was successfully induced by the destruction of cochlear hair cells, which is consistent with the results of previous studies (Hu et al., 2017; Ye et al., 2019). Apoptotic activities in hair cells were significantly upregulated within the following 7 days, whereas no change was seen in SGNs (Figure 1A). The structure of the organ of Corti was almost completely collapsed and destroyed, and nearly no sensory epithelial cells remained in the middle turn on the 30th day after kanamycin and furosemide injection, while almost only a continuous layer of flattened cubic epithelial cells remained on the basilar membrane (Figure 2B). A time-series evaluation of SGN density was conducted on the 7th, 15th, and 30th day after kanamycin and furosemide injection (Figure 1B, auditory physiology data in Supplementary Figure 1 and Supplementary Table 1). On the 30th day after kanamycin and furosemide injection, SGN density has significantly decreased in the apical, middle, and basal region of the cochlea (Welch two sample *t*-test, apical: *t* = 8.4, *P* = 0.00009; middle: *t* = 7.53, *P* = 0.0005; basal: *t* = 10.33, *P* = 0.00003). Thus, this time point was used to decipher the transcriptome changes during SGN degeneration.

### Transcriptome Landscape of Spiral Ganglion Neurons Degeneration

**FIGURE 3:**
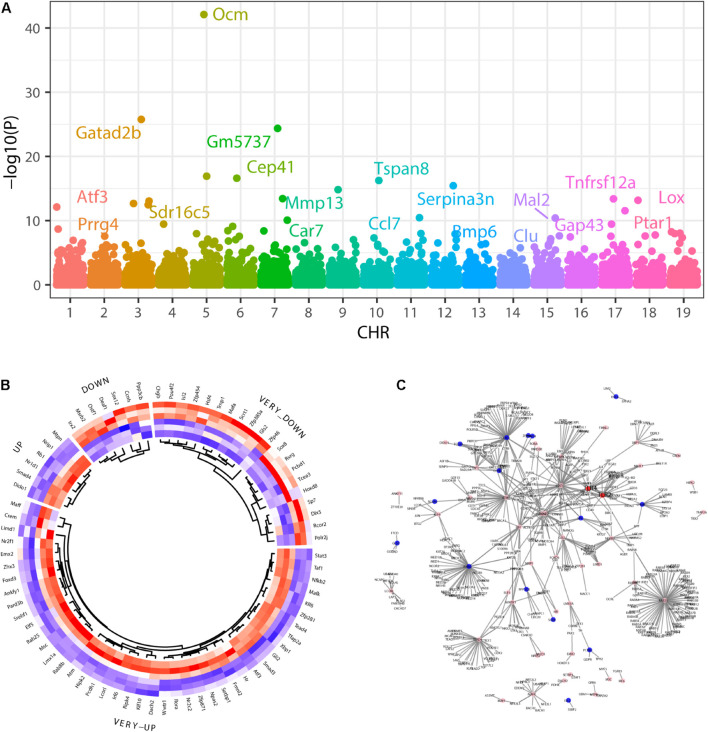
Differentially expressed genes during SGN degeneration. **(A)** Manhattan plot of all genes, including the DEGs. The abscissa indicates the chromosomes, and the ordinate represents the *p*-values of those genes. The most significant gene of each chromosome is labeled. **(B)** Heatmap of the top upregulated and downregulated genes. Blue indicates relatively lower expression, and red indicates relatively higher expression. **(C)** The disturbance of Tlr2/4 on the SGN degeneration network. Blue indicates transcription factors with relatively downregulated expression, and pink indicates relatively upregulated expression. DEGs: differentially expressed genes.

We identified 674 upregulated and 390 downregulated genes in a background of 34147 non-significant genes during SGN degeneration (Supplementary Table 1). Among them, 972 differentially expressed genes (DEGs) had clear autosome positions, and chromosomes 1–19 had 60, 73, 52, 60, 76, 63, 60, 52, 58, 29, 65, 35, 30, 44, 52, 53, 42, 29, and 39 DEGs, of which the most significant ones were Atf3, Prrg4, Gatad2b, Sdr16c5, Ocm, Gm5737, Cep41, Car7, Mmp13, Tspan8, Ccl7, Serpina3n, Bmp6, Clu, Mal2, Gap43, Tnfrsf12a, Lox, and Ptar1 (Figure 3A). Among the differentially expressed genes, there were 76 genes encoding transcription factors (Figure 3B), of which the 10 most significant were Atf3, Stat3, Chgb, Irf6, Scrt1, Hipk2, Zfp871, Rxrg, Lmx1a, and Setbp1.

**FIGURE 4:**
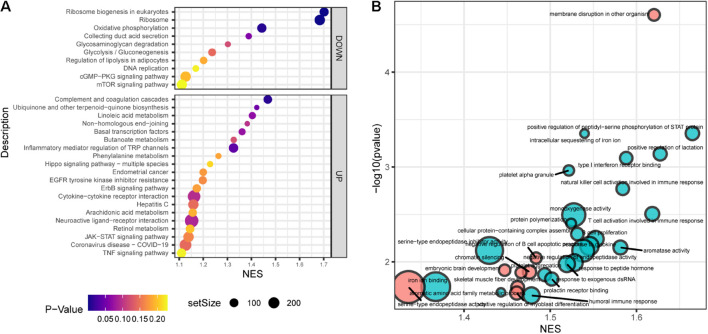
Functional annotation of SGN degeneration. **(A)** KEGG pathway enrichment analysis of SGN degeneration. The abscissa represents the enrichment scores, and the ordinate suggests the KEGG terms. **(B)** GO enrichment analysis of SGN degeneration. The abscissa represents the enrichment scores, and the ordinate shows the –lg (*p*-value). Red: downregulated terms; Blue: upregulated terms.

Gene set enrichment analysis (GSEA) showed that the top30 significantly enriched KEGG pathways included 20 upregulated pathways (Figure 4A), including complement and coagulation cascades, ubiquinone and other terpenoidquinone biosynthesis, linoleic acid metabolism, non-homologous end-joining, basal transcription factors, butanoate metabolism, inflammatory mediator regulation of TRP channels, phenylalanine metabolism, Hippo signaling pathway, endometrial cancer, EGFR tyrosine kinase inhibitor resistance, ErbB signaling pathway, cytokine-cytokine receptor interaction, hepatitis C, arachidonic acid metabolism, neuroactive ligand-receptor interaction, retinol metabolism, JAK-STAT signaling pathway, coronavirus disease (COVID-19), and TNF signaling pathway. The other 10 downregulated KEGG pathways (Figure 4A) were ribosome biogenesis, ribosome, oxidative phosphorylation, collecting duct acid secretion, glycosaminoglycan degradation, glycolysis/gluconeogenesis, regulation of lipolysis in adipocytes, DNA replication, cGMP-PKG signaling pathway, and mTOR signaling pathway. Autophagy and mTOR signaling pathways have been validated to regulate SGN degeneration in our previous work (Ye et al., 2019), which is consistent with the transcriptome results.

GO analysis showed that the dysregulated functions included positive regulation of peptidyl-serine phosphorylation of STAT protein, type I interferon receptor binding, T cell activation involved in immune response, B cell proliferation, natural killer cell activation involved in the immune response, membrane disruption, negative regulation of B cell apoptosis, and embryonic brain development (Figure 4B). The analysis of PPI interactions indicated six key modules (Table 1), and 10 molecules were the most significant, including COMP, COL1A1, STAT3, HAPLN1, HBEGF, CCL2, MMP13, SMAD4, CXCL1, and CTSK. We noticed that these enriched activities of immune responses, TNF signaling pathway, and cytokine-cytokine receptor interaction could be regulated by Tlr molecules (Peek et al., 2020). At the same time, Tlr2 and Tlr4 disrupted the SGN degeneration signaling network (Figure 3C). Based on these results, we hypothesized that Tlr might contribute to the regulation of SGN degeneration.

**TABLE 1:** Key modules of SGN degeneration in WT mice.

Modules	Scores	No.	Edges/Interactions	Gene name
1	4.222	10	27	*COMP, COL1A1, STAT3, HAPLN1, HBEGF, CCL2, MMP13, SMAD4, CXCL1, CTSK*
2	4	7	14	*SNAP25, SYN2, NGFR, CALB2, GAP43, NEFH, SYP*
3	3.667	7	12	*LOX, ITPKB, CDH1, SMAD3, EGF, CTGF, TIMP2*
4	3.2	6	10	*MMP9, COL4A1, SDC4, CCL12, CCL7, VCL*
5	3	3	4	*SDR16C5, PLAG1, LCORL*
6	2.8	6	10	*CALM3, TMOD4, TPM4, CALM2, TCAP, PNCK*

### Tlr2/4 Double Knockout Mice Has Auditory Advantage in Spiral Ganglion Neurons Degeneration

Given that Tlr may play an important role in SGN degeneration, we established Tlr2 and Tlr4 knockout mice. We detected the hearing threshold of Tlr2/4 DKO mice and wild-type littermates before and 30 days after SGN degeneration. The Tlr DKO group of mice treated with normal saline (NS) and the wild type (WT) mice maintained normal hearing thresholds (Figure 2A), and there was no significant difference in the hearing thresholds of the two groups of mice (*P* = 0.748, *F* = 0.105, two-way ANOVA). The Tlr DKO mice of the SGN degeneration group exhibited hearing threshold advantages (*P* = 0.00446, *F* = 8.700, two-way ANOVA) on the 30th day after kanamycin and furosemide injection, and the hearing preservation advantage was mainly manifested in 4–16 kHz (4 kHz, *t* = –3.16, *P* = 0.012; 8 kHz, *t* = –3.12, *P* = 0.0012; 11 kHz, *t* = –2.51, *P* = 0.041, 16 kHz, *t* = –3.77, *P* = 0.0059; 22 kHz, *t* = –1.44, *P* = 0.19, 32 kHz, *t* = *–*0.22, *P* = 0.83; *N* = 17, Welch Two Sample *t*-test) (Figure 2A and Supplementary Table 1).

We also observed morphological changes in the Organ of Corti and spiral ganglion neurons. The Tlr2/4 DKO mice and their wild-type littermates treated with normal saline (NS) maintained relatively intact inner and outer hair cells, while the Tlr DKO mice and their wild-type littermates under the SGN degeneration model lost most inner and outer hair cells, and the tunnel of Corti collapsed (Figure 2B).

The Tlr DKO mice and their wild-type littermates treated with NS maintained a relatively normal density, with no significant difference in neuron density (*P* = 0.675, *t* = 0.43, *N* = 24, Welch Two Sample *t*-test). SGN density significantly decreased in Tlr DKO mice and wild-type mice under the SGN degeneration model (Figure 2C), but the SGN density was significantly higher in the DKO group mice (*P* < 0.001, *t* = 11.71, *N* = 32, Welch Two Sample *t*-test) (Figure 2D). These results suggest that the hearing preservation advantage of Tlr2/4 DKO mice mainly relies on the protection of spiral ganglion neurons.

### Inverted Gene Set Enrichment of Double Knockout Mice During Spiral Ganglion Neurons Degeneration

The p38-mediated MAPK and p65-mediated NF-κB signaling pathways are canonical signaling pathways of Tlr molecules (Li et al., 2010). We used immunohistochemistry to detect the expression of these genes in SGNs. SGN p38 expression was found in both DKO and WT mice, mainly in the cytoplasm and nuclei. There was no significant difference in p38 expression between the two groups on the 30th day of degeneration induction (Figure 2E, neuron density evaluation in Supplementary Figure 2). The SGNs of DKO and WT mice express p65 (RelA), which is mainly located in the cytoplasm and nuclei. P65 expression decreased 30 days after SGN degeneration, but no significant differences in expression were identified between the two groups (Figure 2F, neuron density evaluation in Supplementary Figure 2).

**FIGURE 5:**
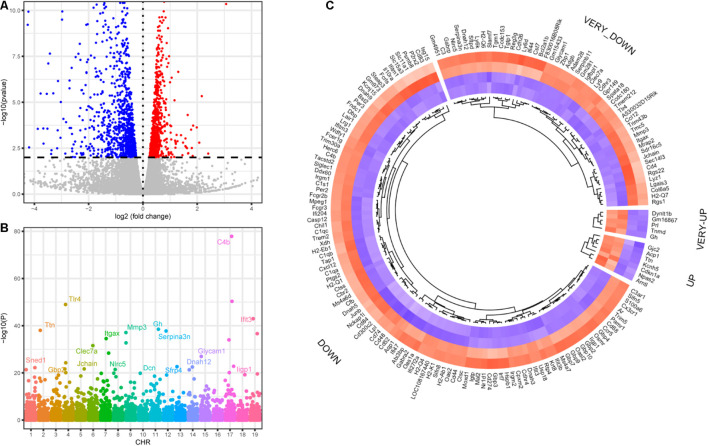
Differentially expressed genes of DKO VS. WT SGN transcriptome on the 30th day. **(A)** Volcano plot of all detected genes including the DEGs. Red dots indicate upregulated genes and blue dots indicate downregulated genes. **(B)** Manhattan plot of all genes including the DEGs. The abscissa indicates the chromosomes, and the ordinate represents the *p*-values of those genes. The most significant gene of each chromosome is labeled. **(C)** Heatmap of the top upregulated and downregulated transcription factors. If the log_2_ fold change is higher than 2, the category is labeled with VERY. Blue indicates relatively lower expression, and red indicates relatively higher expression.

Transcriptomic analysis was performed on the modiolus of DKO and WT mice on the 30th day after kanamycin and furosemide administration. We identified 1029 upregulated genes and 833 downregulated genes with the criteria of *P* < 0.01, and | log_2_ fold change | > 0.2) (Figure 5A; Supplementary Table 1). Among them, 1,747 had a clear autosomal location and there were 136, 133, 86, 103, 125, 107, 126, 82, 96, 80, 149, 70, 65, 78, 76, 56, 75, 39, 65 on chromosome 1–19, respectively. The most strongly differentially expressed genes on autosomal chromosomes were identified, including Sned1, Ttn, Gbp2, Tlr4, Jchain, Clec7a, Itgax, Nlrc5, Mmp3, Dcn, Gh, Serpina3n, Sfrp4, Dnah12, Glycam1, Parp14, C4b, Iigp1, and Ifit3 (Figure 5B). Figure 5C shows the differentially expressed transcription factors.

**FIGURE 6:**
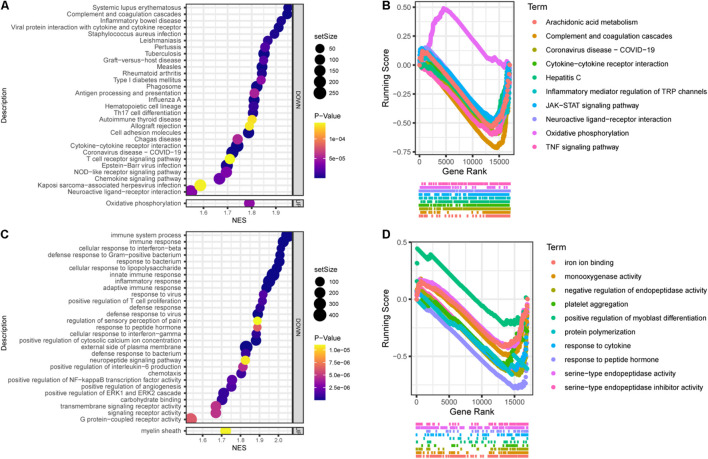
Functional annotation of DKO VS. WT SGN transcriptome on the 30th day. **(A)** KEGG pathway enrichment analysis. The abscissa represents the enrichment scores, and the ordinate suggests KEGG terms. **(B)** The running score visualization of the top 10 enriched KEGG terms which are also involved in the SGN degeneration process. **(C)** GO enrichment analysis of SGN degeneration. The abscissa represents the enrichment scores, and the ordinate suggests KEGG terms. **(D)** The running score visualization of the top 10 enriched KEGG terms which are also involved in SGN degeneration process.

In general, Tlr2/4 DKO mice showed downregulated biological activities during SGN degeneration compared to WT mice. The top 30 enriched KEGG pathways included 1 upregulated and 29 downregulated pathways (Figure 6A). Oxidative phosphorylation was downregulated during SGN degeneration, but upregulated in DKO mice compared with WT mice, which is called inverted gene set enrichment. The top 10 KEGG terms with inverted gene set enrichments included arachidonic acid metabolism, complement and coagulation cascades, coronavirus disease—COVID-19, cytokine-cytokine receptor interaction, hepatitis C, inflammatory mediator regulation of TRP channels, JAK-STAT signaling pathway, neuroactive ligand-receptor interaction, TNF signaling pathway, and oxidative phosphorylation (Figure 6B).

Similarly, the top 30 enriched GO terms included one upregulated and 29 downregulated activities (Figure 6C). The top 10 KEGG terms with inverted gene set enrichments included iron ion binding, monooxygenase activity, negative regulation of endopeptidase activity, platelet aggregation, positive regulation of myoblast differentiation, protein polymerization, response to cytokines, response to peptide hormones, serine-type endopeptidase activity, and serine-type endopeptidase inhibitor activity (Figure 6D).

### Suspectable Core Modules That Attenuate Spiral Ganglion Neurons Degeneration in Double Knockout Mice

In order to detect whether DKO mice had consistent morphology across the entire signaling network, i.e., whether there is an inverted gene set enrichment relationship with the primary auditory neurons degeneration (PAND)-specific gene set, we analyzed the different genes in the PAND-specific gene set. We found that there was an inverted GSEA relationship between the PAND process and the PAND-specific gene set in DKO mice, and the PAND-associated DEGs were relatively reverse regulated in the progression of PAND in DKO mice (NES = –1.84, *P* < 0.001).

We further analyzed the attenuation of SGN degeneration in DKO mice from the perspective of the entire signaling network (Supplementary Figure 3). We found that 130 of the 661 genes specifically upregulated during the degeneration of SGN in WT mice were downregulated mice during the degeneration of spiral neurons in DKO mice, and 380 genes were specifically downregulated during the degeneration of SGN in WT mice; 116 genes were downregulated during the degeneration of spiral neurons in DKO mice.

Protein interaction analysis of the proteins encoded by these genes showed that DKO mice had a specific anti-degenerative signal network compared to WT mice. The disease module analysis showed that there were two key modules (Table 2). The score of the first module was 3.667, including seven genes, namely *EGF, STAT3, CALB2, LOX, SNAP25, CAV2*, and *SDC4*. The score of the second module was 3.5, which included five genes: *MYL1, NCS1, PVALB, TPM4, and TMOD4. In addition, EGF, STAT3, CALB2, SNAP25, SDC4, TPM4*, and *TMOD4* are key molecules involved in SGN degeneration. CALB2 is a specific marker of the SGN Ia and Ib subtypes in mice (Shrestha et al., 2018), PVALB is a neuron-specific marker (Petitpré et al., 2018), and the interaction of these molecules may contribute to the attenuation of SGN degeneration in DKO mice.

**TABLE 2:** Key modules of the attenuation of SGN degeneration in DKO mice.

Modules	Score	No.	Edges/interactions	Gene name
1	3.667	7	12	*EGF, STAT3, CALB2, LOX, SNAP25, CAV2, SDC4*
2	3.5	5	9	*MYL1, NCS1, PVALB, TPM4, TMOD4*

## Discussion

Studies have shown that primary and secondary SGN degeneration may account for half of the SGN degeneration (Makary et al., 2011; Waqas et al., 2017; Guo et al., 2021). Morphological studies of the human cochlea show that with the growth of age, even if there is no loss of inner and outer hair cells, SGN will be lost at a rate of about 100 cells per year. This rate can increase to approximately 185 cells per year when there is simultaneous loss of inner and outer hair cells (Makary et al., 2011). Unlike auditory hair cells, which are easily damaged by noise and ototoxic drugs such as aminoglycosides and cisplatin, spiral ganglion neurons are relatively robust. In a study of animal models and human temporal bone, histopathological analysis of acquired sensorineural hearing loss showed that severe degeneration of cochlear spiral neurons is often accompanied by a significant loss of hair cells (Miller et al., 1997; McFadden et al., 2004). The degeneration of SGNs seems to be related to the loss of inner hair cells, because 95% of spiral neurons only have synaptic connections with inner hair cells. However, the causal relationship based on histopathological correlation is often frustrated (Zilberstein et al., 2012), which may be because the loss of inner hair cells is usually accompanied by the loss or complete destruction of the organ of Corti after cochlear injury, and both hair cells and supporting cells contribute to the support of SGN in the cochlea (Sugawara et al., 2005). Temporal bone studies from 54 to 89 years of age without ear diseases suggest that the number of hair cells is almost normal, but the pattern of axonal degeneration of the cochlear nerve may be an important form of human presbycusis (Viana et al., 2015).

Transcriptome analysis of age-related cochlear degeneration indicated that no immune or inflammatory activity was enriched (Supplementary Figure 4). Therefore, the present study utilized the secondary SGN degeneration model of a single dose injection of kanamycin and furosemide, and the SGN degeneration model is suitable for the study of Tlr regulation during SGN degeneration. In the present SGN degeneration model, almost all outer hair cells were destroyed within 7 days after the injection of kanamycin and furosemide. Inner hair cells seemed more robust and about 15% inner hair cells were preserved 30 days after the treatment. In other words, <5% hair cells (including OHCs and IHCs) remain alive on the 30 day (Hu et al., 2017; Ye et al., 2019). These results were also in consistent with the study of Taylor (Taylor et al., 2008).

Another important question is when this transcriptome changes happen in DKO mice. We consider that the key points might be the opposite regulated direction of DKO mice compared with a normal SGN degeneration transcriptome change. In other words, the overlapping and opposite terms would be highlighted that might play key role in the alleviation of SGN degeneration in DKO mice whenever it happened. But we should acknowledge that it is preferred if these changes occurred after the degeneration induction, otherwise a systematic influence and specific delivery of these molecules should be considered. Tlr-regulated innate immune signaling regulates neuron-glia interactions (McLaughlin et al., 2019). McLaughlin et al. demonstrated that glia non-canonical Toll-like receptor signaling could be non-autonomously activated by neuronal apoptosis, priming their capacity to engulf apoptotic neurons and regulate the maintenance of a healthy brain (Kashio and Miura, 2019). Glia continuously surveys neuronal health during development, providing trophic support to healthy neurons, while rapidly engulfing dying ones. With neuronal health being surveyed, glia could provide trophic support to healthy neurons and rapidly engulf dying neurons. Glia necessitates a foolproof mechanism to unambiguously identify those neurons to support vs. engulf. To ensure specificity, glia is proposed to interact with dying neurons via a series of carefully choreographed steps. Dying neurons and glia communicate via toll-receptor-regulated innate immune signaling, while neuronal apoptosis drives the processing and activation of the Toll-6 ligand and activates the dSARM-mediated Toll-6 transcriptional pathway, which controls the expression of the Draper engulfment receptor. Pathway loss drives early onset neurodegeneration, underscoring its functional importance.

Zhang et al. (2019) demonstrated that in the cochleae (lateral wall and spiral ganglion neurons), TLR-4 and the downstream signaling molecule MyD88 were significantly upregulated 3 d after noise exposure. It has also been reported that Tlr4 knockout mice could inhibit the expression of major histocompatibility complex class II and participate in the antigen-presenting function of macrophages after acoustic trauma (Vethanayagam et al., 2016). Their results suggested that Tlr4 regulates multiple aspects of the immune response in the cochlea and contributes to cochlear pathogenesis after acoustic injury. According to the single-cell transcriptome of Shrestha et al. (2018) and our IHC results (data not shown), SGN may rarely be Tlr2/4 positive. Tlr2/4 double knockout may attenuate SGN degeneration by disturbing the local microenvironment of circular macrophage cells or Schwann cells and detecting Tlr downstream in SGN fails to signify the attenuation of SGN degeneration.

We identified some relatively novel molecules that may play an essential role in attenuating SGN degeneration in Tlr2/4 DKO mice. Researchers have found that the interaction between Tlr and these molecules may regulate the pathogenesis of many diseases. For example, myofibroblast Tlr signaling promotes colitis-associated carcinogenesis by mediating macrophage M2 polarization and STAT3 activation via intracellular communication (Yuan Q. et al., 2021, p. 88). Hsu et al. (2010) found that the differentially regulated expression of Tlr-associated epidermal growth factor influences mucosal healing.

DFNA44, an autosomal dominant deafness, is tightly associated with the effector of EGF-mediated cell signaling (Modamio-Hoybjor et al., 2007). Rats administered oral epigallocatechin-3-gallate demonstrated reduced cisplatin-induced hearing loss, experienced reduced loss of OHCs in the basal region of the cochlea and reduced oxidative stress and apoptotic markers. The preservation of STAT3 and Bcl-xL activation and increased STAT3/STAT1 ratio may contribute to protection (Borse et al., 2017). STAT3 and its activated form are specifically expressed in hair cells during cochlear development (Chen et al., 2017). CALB2 is a candidate gene for human dominant optic atrophy (Carelli et al., 2011, p. 8). Calb2 is highly expressed in the Ia and Ib subtypes of SGN (Shrestha et al., 2018; Sun et al., 2018). In noise-induced hearing loss (NIHL), noise exposure damages cochlear sensory hair cells, which lack the capacity to regenerate. Following noise insult, intense metabolic activity occurs, resulting in a free-radical imbalance in the cochlea. Oxidative stress and antioxidant enzyme alterations, including lipoxygenase (LOX) upregulation, have been linked to chronic inflammation, which contributes to hearing impairment. Inhibition of LOX showed greater efficacy in the treatment of NIHL (Rodriguez et al., 2017). LOX products may contribute to the hypersensitivity of SGN to hair cell inputs in a variety of pathological conditions (Balaban et al., 2003). Snap25 is a key marker of neuronal identity. Noda et al. (2018) used the neurogenic pioneer transcription factor Ascl1 and the auditory neuron differentiation factor NeuroD1 to reprogram spiral ganglion non-neuronal cells into induced neurons. Their transcriptome results indicated that induced neurons maintained some key markers of neuronal identity, such as Tubb3, Map2, Prph, Snap25, and Prox1 (Noda et al., 2018). The mutation of the otoferlin C2 domain, which causes deafness in humans, impairs the ability of otoferlin to bind syntaxin, SNAP-25, and the Cav1.3 calcium channel, which may mediate regulation by otoferlin of hair cell synaptic exocytosis, which is critical to inner ear hair cell function (Ramakrishnan et al., 2009). The damaged interaction between SDC4 and GIPCs also contributes to the progression of non-syndromic hearing loss (Katoh, 2013).

## Conclusion

In conclusion, our results suggest that the SGN degeneration of Tlr2/4 DKO mice and their wild-type littermates exhibits an inverted GSEA relationship, and DKO mice may attenuate SGN degeneration by the differential regulation of some core molecules, including EGF, STAT3, CALB2, LOX, SNAP25, CAV2, SDC4, MYL1, NCS1, PVALB, TPM4, and TMOD4.

## Data Availability Statement

The datasets presented in this study can be found in online repositories. The names of the repository/repositories and accession number(s) can be found below: NCBI Gene Expression Omnibus (GEO) under accession number GSE182978.

## Ethics Statement

The animal study was reviewed and approved by the Ethics Committee of Xinhua Hospital, affiliated with Shanghai Jiao Tong University School of Medicine.

## Author Contributions

MX and QW were responsible for the initiation and conduction, respectively. QW was responsible for writing the manuscript. QW, YS, YP, TZ, AZ, DG, CF, HH, LM, KC, and BY performed the experiments. BY and QW processed and analyzed the data. All authors contributed to the article and approved the submitted version.

## Conflict of Interest

The authors declare that the research was conducted in the absence of any commercial or financial relationships that could be construed as a potential conflict of interest.

## Publisher’s Note

All claims expressed in this article are solely those of the authors and do not necessarily represent those of their affiliated organizations, or those of the publisher, the editors and the reviewers. Any product that may be evaluated in this article, or claim that may be made by its manufacturer, is not guaranteed or endorsed by the publisher.

## Supplementary Material

Supplementary Figure 1 The hearing threshold of auditory brainstem response of WT mice in the SGN degeneration model. Mice maintained normal hearing threshold in the normal saline treated group. On the 30th day after kanamycin and furosemide injection, mice exhibited a hearing deteriation compared with NS treated mice (*N* = 13, *P* < 0.00001, *F* = 275.910, two-way ANOVA).

Supplementary Figure 2 Reconfirmation of SGN density of sections with histopathological analysis. The upper panel for p38 and the lower panel for p65.

Supplementary Figure 3 Protein-protein interaction network constructed DKO VS. WT SGN transcriptome on the 30th day. Red: upregulated molecules. Blue: downregulated molecules.

Supplementary Figure 4 Differentially expressed genes during whole cochlear aging. **(A)** Manhattan plot of all genes, including the DEGs. The abscissa indicates the chromosomes, and the ordinate represents the *p*-values of those genes. The most significant gene of each chromosome is labeled. **(B)** Heatmap of the top upregulated and downregulated genes. Blue indicates relatively lower expression, and red indicates relatively higher expression. **(C)** KEGG pathway enrichment analysis. The abscissa represents the enrichment scores, and the ordinate suggests –lg (*p*-value).
