# Supplementary material for: Tlr2/4 Double Knockout Attenuates the Degeneration of Primary Auditory Neurons: Potential Mechanisms From Transcriptomic Perspectives
Source: Front Cell Dev Biol. 2021 Oct 25;9:750271. doi: 10.3389/fcell.2021.750271 (PMC8573328; doi:10.3389/fcell.2021.750271)
Supplement: Supplementary Figure 1 — The hearing threshold of auditory brainstem response of WT mice in the SGN degeneration model. Mice maintained normal hearing threshold in the normal saline treated group. On the 30th day after kanamycin and furosemide injection, mice exhibited a hearing deteriation compared with NS treated mice (N = 13, P < 0.00001, F = 275.910, two-way ANOVA). [file Data_Sheet_1.PDF]

frontiers

Supplementary Material

1 Supplementary Figures

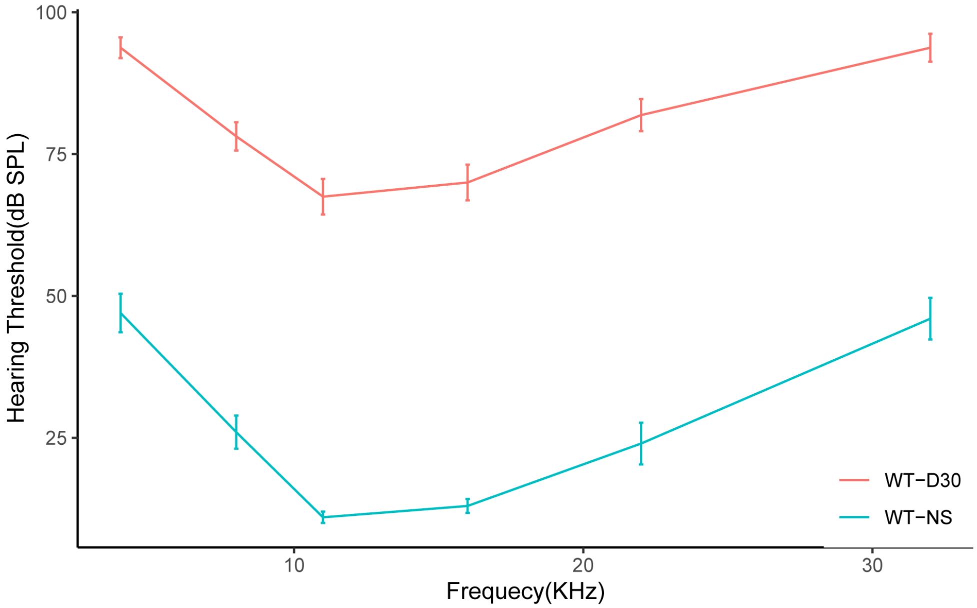

Supplementary Figure 1. The hearing threshold of auditory brainstem response of WT mice in the SGN degeneration model. Mice maintained normal hearing threshold in the normal saline treated group. On the 30th day after kanamycin and furosemide injection, mice exhibited a hearing deteriation compared with NS treated mice ($N = 13$, $P < 0.00001$, $F = 275.910$, two-way ANOVA).

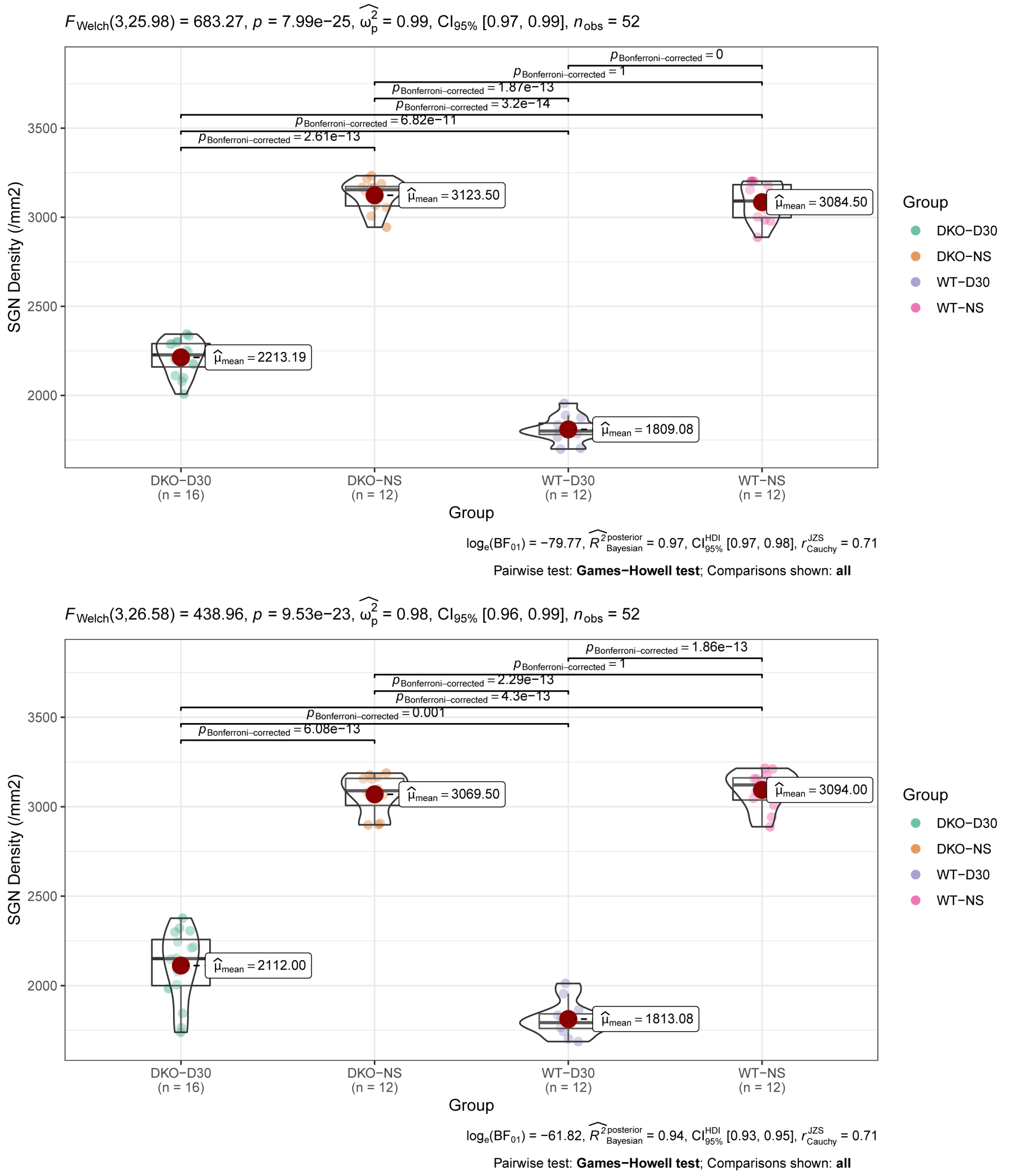

Supplementary Figure 2. Reconfirmation of SGN density of sections with histopathological analysis. The upper panel for p38 and the lower panel for p65.

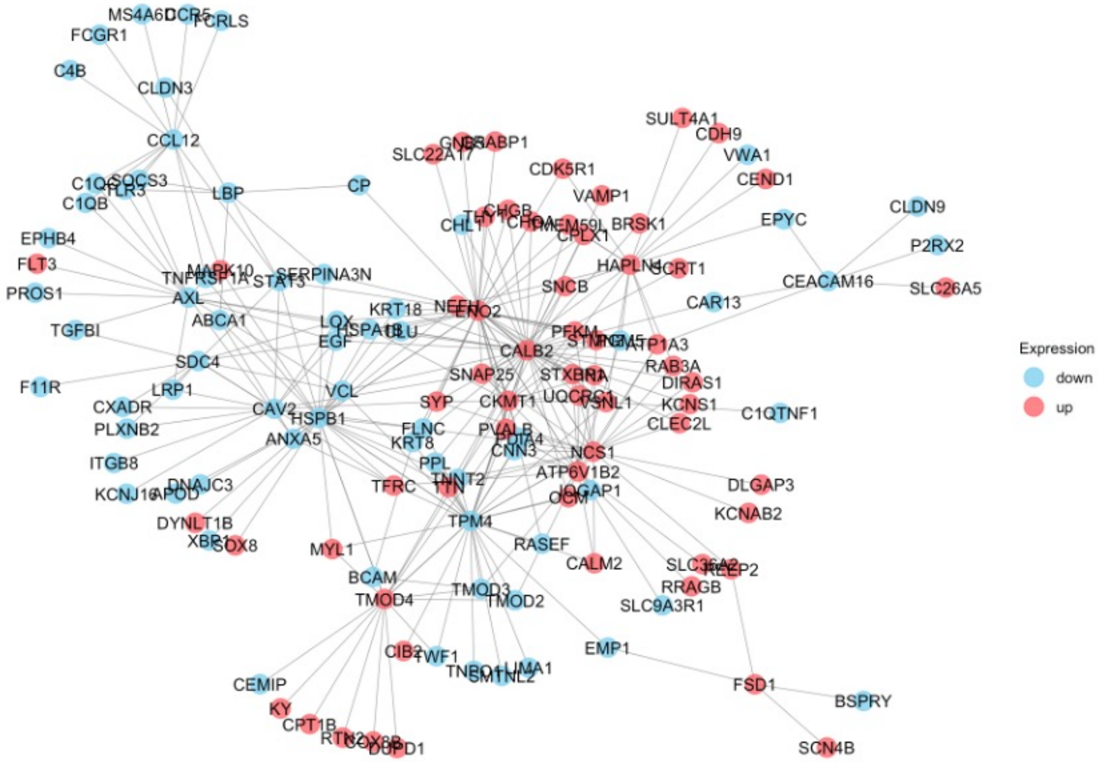

Supplementary Figure 3. Protein-protein interaction network constructed DKO VS. WT SGN transcriptome on the 30th day. Red: upregulated molecules. Blue: downregulated molecules.

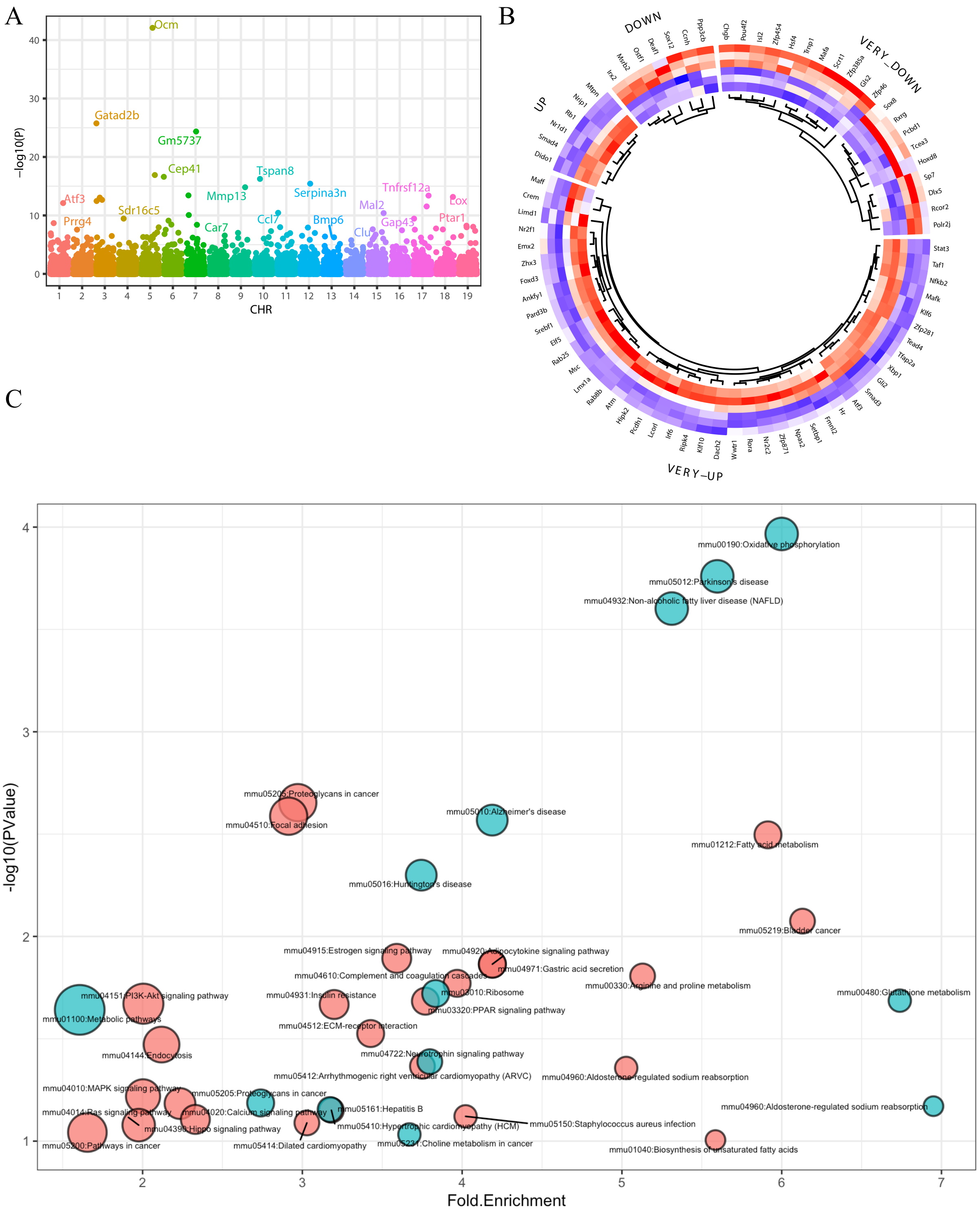

Supplementary Figure 4. Differentially expressed genes during whole cochlear aging (Data: NCBI GSE35234). (**A**) Manhattan plot of all genes, including the DEGs. The abscissa indicates the chromosomes, and the ordinate represents the p values of those genes. The most significant gene of each chromosome is labeled. (**B**) Heatmap of the top upregulated and downregulated genes. Blue indicates relatively lower expression, and red indicates relatively higher expression. (**C**) KEGG

pathway enrichment analysis. The abscissa represents the enrichment scores, and the ordinate suggests -lg(p-value).
